# Supplemental Nutrition Assistance Program-Education (SNAP-Ed) Supporting Innovative Nutrition Education for American Indian and Indigenous-Serving Organizations in Colorado

**DOI:** 10.1016/j.cdnut.2024.104492

**Published:** 2024-10-29

**Authors:** Sarah A Stotz, Kelli Begay, Shannon Francis, Devon Peña, Spero M Manson, Jini E Puma

**Affiliations:** 1 Department of Food Science and Human Nutrition, Colorado State University, Fort Collins, CO, United States; 2 Maven Collective Consulting, LLC, Albuquerque, NM, United States; 3 Spirit of the Sun, INC, Denver, CO, United States; 4 Department of American Ethnic Studies, Department of Anthropology, University of Washington, Acequia Institute, San Luis, CO, United States; 5 Centers for American Indian and Alaska Native Health, Colorado School of Public Health, University of Colorado Anschutz Medical Campus, Aurora, CO, United States; 6 Rocky Mountain Prevention Research Center, Colorado School of Public Health, University of Colorado Anschutz Medical Campus, Aurora, CO, United States

**Keywords:** nutrition education, American Indian, Indigenous, SNAP-Ed, federal food assistance program

## Abstract

**Background:**

Supplemental Nutrition Assistance Program (SNAP)-Education (Ed) is the nutrition education component of the United States Department of Agriculture (USDA) SNAP.

**Objectives:**

The objective of this study was to elucidate the experiences of 3 CO-based, American Indian, or Indigenous-led organizations who engaged in a SNAP-Ed pilot funding project in fiscal year 2022.

**Methods:**

An instrumental, single case study design was employed to conduct this evaluation. The multiphase method evaluation included listening sessions (*n* = 8 Indigenous-serving organizations), invited requests for proposals, pilot award funding (*n* = 3 awarded organizations), and longitudinal, storytelling-based qualitative data collection during the funding cycle.

**Results:**

There are many ongoing food and nutrition education programs serving Indigenous/American Indian peoples in CO, which largely promote wellness and healing through traditional cultural practices and are not overtly framed as “nutrition education.” Two salient themes emerged: *1*) defining and describing indigenized, decolonized nutrition education, and *2*) description of experiences working with SNAP-Ed funding during FY2022, including challenges and future opportunities.

**Conclusions:**

To effectively address the priority area for SNAP-Ed funding to reach Tribal communities in each state, understanding the perspectives of Tribal and Indigenous-led communities who are new to SNAP-Ed funding is prudent. Next steps to continued cocreation of SNAP-Ed opportunities in Indigenous spaces include educating funders and policymakers on Indigenous ways of knowing, traditional food and nutrition practices, and importance of relationality, land, healing, and other Indigenous values. Determining the “success” of a program (e.g., program evaluation) needs to be defined by the community itself.

## Introduction

Supplemental Nutrition Assistance Program (SNAP)-Education (Ed) is the nutrition education component of the USDA SNAP, formerly known as Food Stamps. It is funded through the Nutrition Education and Obesity Prevention Grant Program in the Healthy Hunger-Free Kids Act of 2010 [1]. The key goal of SNAP-Ed is to improve the likelihood that people with incomes at or <185% of the federal poverty level, especially those residing in communities with a significant low-income population, to make healthy food choices within a limited budget and choose physically active lifestyles consistent with the Dietary Guidelines for Americans [2]. The SNAP State Agencies in all 50 states, the District of Columbia, Guam, and the United States Virgin Islands participate in SNAP-Ed by contracting with 164 SNAP-Ed State Implementing Agencies to conduct health promotion programming [3]. Many state-implementing agencies subcontract different types of public, nonprofit, and business organizations to provide direct services. Collectively, hundreds of interventions tailored to Americans with low income of different age, race/ethnicity, and linguistic segments are delivered each year in compliance with approved state plans and the SNAP-Ed Plan Guidance of the Food and Nutrition Service, an agency of the USDA.

The SNAP-Ed Plan Guidance defines nutrition education and prevention obesity services broadly as “a combination of educational strategies, accompanied by supporting policy, systems, and environmental interventions, demonstrated to facilitate adoption of food and physical activity choices and other nutrition-related behaviors conducive to the health and well-being of SNAP participants and low-income individuals eligible to receive benefits under SNAP or other means-tested programs and individuals residing in communities with a significant low-income population” [4]. As such, this includes funding for activities such as cooking classes, gardening workshops, nutrition classes, food acquisition classes/workshops (e.g., growing and harvesting), and food preservation classes/workshops (e.g., canning and drying). Other examples include farmers market or grocery store tours, school-based hands-on cooking classes for children, and social media-based education. Other SNAP-Ed-funded initiatives include working with grocery stores or small corner stores to improve the healthy options available and provide signage. Finally, some SNAP-Ed initiatives focus on helping to create or sustain policies that promote health and well-being, such as eliminating vending machines in schools or safe walking routes to schools.

Throughout its history, SNAP-Ed has evolved to emphasize inclusivity and equality in representation. Clearly detailed in the Federal Fiscal Year (FY) 2024 SNAP-Ed Plan Guidance is the requirement that “State agencies consult with Tribes about the SNAP State Plan of Operations, which includes the State SNAP-Ed Plan. State agencies must actively engage in timely, meaningful, and substantive dialog with Tribal leadership or their designees, as required by SNAP regulations at 7 CFR 272.2(b), 272.2(e)(7), and 7 CFR 281.2(b). State agencies must consider and include the needs of American Indian/Alaska Native (AI/AN) populations in conducting a holistic needs assessment for SNAP-We and coordinate with State and local operators on how those needs assessments can be conducted”[4,5]. In CO, the Colorado Department of Human Services serves as the State Agency for SNAP-Ed in CO and will conduct official Tribal consultation with the Southern Ute Indian Tribe in Ignacio, CO, and the Ute Mountain Ute Tribe in Towaoc, CO. In advance of this new SNAP-Ed plan requirement and to begin building meaningful partnerships, Colorado SNAP-Ed prioritized supporting Indigenous communities in addition to formal Tribal consultation and collaboration with the Tribes through SNAP-Ed funding.

The purpose of the present study was to elucidate the experiences of 3 CO-based, Indigenous-led organizations that engaged in a SNAP-Ed pilot funding project in FY 2022. The research question was: “What is the experience of Indigenous-serving organizations that utilize SNAP-Ed funding through a pilot funding mechanism?” To answer this research question, Colorado SNAP-Ed leadership partnered with staff and faculty from the University of Colorado Anschutz Medical Campus – Centers for American Indian and Alaska Native Health (CAIANH). Information gained from answers to this research question is important to advance science using community-engaged methods to enhance equity and inclusion of public health nutrition programming. Of note, throughout this manuscript, we have selected to use the term “Indigenous” to be inclusive of Native American, American Indian, and Indigenous people of Mexican origin who reside in what is now known as the United States, per the preference of the majority of the Indigenous authors on the paper.

## Methods

### Theoretical framework

This case study is theoretically supported by a constructivist approach [6], drawing upon Traditional Ecological Knowledge and Ancestral Knowledge Systems [7]. The social constructivist perspective suggests that people are continuously developing meanings and understandings in social, cultural, and historical contexts. From this perspective, people construct, form, and negotiate subjective, complex understandings of food, eating, and health through their personal experiences and interactions with other people and their contextual environment [8]. Concepts from both the Traditional Ecological Knowledge and Ancestral Knowledge Systems support Indigenizing research, redistributing power to traditionally marginalized communities, honoring and centering Indigenous ways of knowing and values, and challenging hegemonic Western research practices with critical consciousness [9]. Together, these frameworks informed our culturally relevant approach to sampling, data collection, and data analysis.

### Methodology: case study design

**Figure 1:**
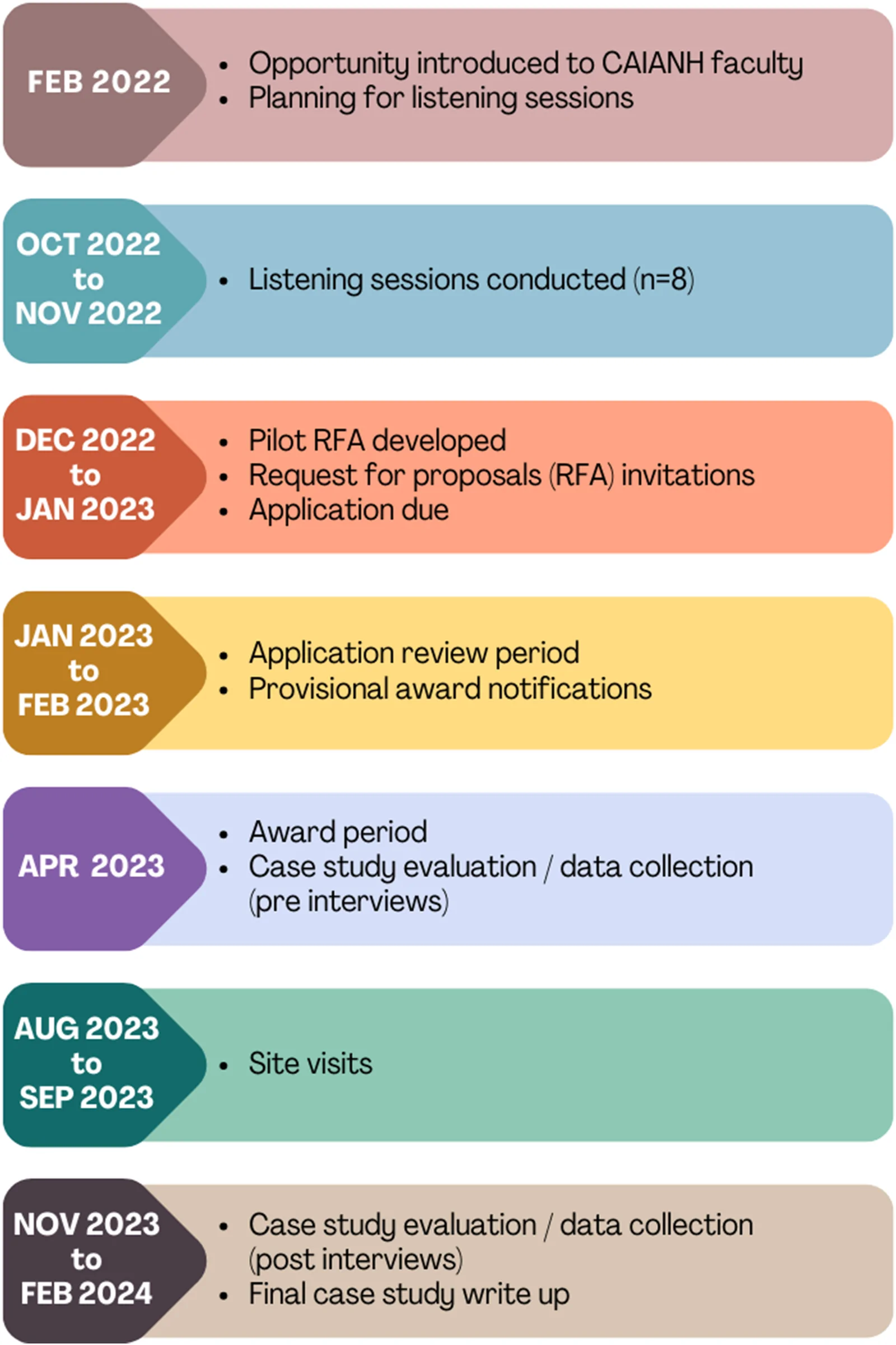
Timeline for Colorado SNAP-Ed in indigenous communities project.

A single case study design was employed to conduct this evaluation [10]. The unit of analysis is defined as the pilot funding mechanism itself. This unit of analysis was selected to tell a comprehensive, rich story across grantees using the unique nature of this funding opportunity. Case study methodology as in-depth formative evaluation of novel program evaluation allows researchers to view problems from multiple perspectives and enriches the meaning of a singular perspective. Two purposes of case study inquiry are to describe an intervention and explore situations where intervention has no single output [10]. This case study is bound by the geographic location of each grantee (e.g., CO) and the 7-mo funding cycle (February 2022–September 2023). Details of the timeline can be found in Figure 1.

To authentically engage CO-based Indigenous communities in SNAP-Ed, the process was 2-fold. First, during phase 1, CAIANH faculty and staff conducted a series of formative evaluation storytelling-based listening sessions [11,12] with CO-based Indigenous-serving organizations that already offer food or nutrition programming. The purpose of these listening sessions was to understand what food and nutrition programs already exist, how SNAP-Ed funding may support these programs, how to best engage Indigenous-serving organizations with SNAP-Ed support, and, most importantly, how to help develop and frame the request for proposal (RFP). Between October–November 2022, 8 such listening sessions were conducted. These sessions were not recorded per preference of the interviewees. Phase 2 of this effort developed an RFP and invited Indigenous-serving organizations that participated in phase 1 (listening sessions) to request pilot funding through the RFP to operationalize existing SNAP-Ed funding to support their relevant, eligible nutrition and food projects through September 2023. Three organizations submitted applications and were awarded funding as requested. This project was determined exempt by the University of Colorado Multiple Institutional Review Board; therefore, informed consent was waived (COMIRB #23-0290). The purpose of this phased approach was to enhance scientific rigor using a community-based participatory approach and to include recommendations and feedback from the priority audience (e.g., listening sessions) when developing the RFP.

### Listening session methods

During phase 1, 1 (non-Indigenous) CAIANH faculty and 1 Indigenous staff member conducted a series of listening sessions with CO-based Indigenous-serving organizations that provide food or nutrition programming. The purpose of these listening sessions was to understand what food and nutrition programs already exist, how SNAP-Ed funding may support these programs, how best to engage Indigenous-serving organizations with SNAP-Ed support, and how to inform the RFP development. Indigenous-serving organizations and the 2 federally recognized Tribes in CO were invited through existing social networks between CAIANH faculty and staff and these organizations. Additionally, organizations were invited to participate in listening sessions through the Colorado Commission of Indian Affairs Health and Wellness Committee quarterly meetings. At 2 of these quarterly meetings, a CAIANH faculty member presented the project and invited those interested in participating in the listening sessions to connect. Invited organizations included urban and rural organizations, Tribes, Tribal organizations, and Indigenous-led/Indigenous-serving organizations across CO. The moderator guide used to facilitate listening sessions can be found in Table 1. Per preference of the listening session participants, the names of the participants and their organization affiliations are withheld.

**TABLE 1:** Moderator guides used for listening sessions and pre-/post Pilot Grantee interviews

Interview Timepoint	Moderator guide questions
Listening sessions with Indigenous-serving organizations in CO Data collected in October – November, 2022 (*n* = 8)	• When you think about wellness and community, what comes to mind? ○ Probes: strengths of community, examples of programming, examples of culture/wellness • Tell me about any existing/current nutrition education and physical activity opportunities in your community. ○ Probes: location, who hosts, attendance, priority audience, frequency, need for funding, cooking classes, gardening workshops • Tell me about any former nutrition education and physical activity opportunities available to your community that either did or didn’t work well with your community. ○ Probes: loss of funding, no participation, barriers to participation • What makes it hard for families to achieve wellness in your community? ○ Probes: lack of fresh produce, expense, places to be physically active • SNAP-Ed defines nutrition and physical activity education quite broadly. This can include things like cooking classes, gardening workshops, farmers market or grocery store tours, school-based hands-on cooking classes for children, social media-based education, working with grocery stores or small corner stores to improve the healthy options available and provide signage, or even helping to create or sustain policies that promote health and well-being: such as no vending machines allowed in schools, safe routes to schools, assisting a neighborhood with wellness changes they would like to see etc. The goal is to make SNAP-Ed work for your community. If you think about nutrition education broadly, what comes to mind that may be beneficial for your community? • Is there anything else on this topic that you’d like to share with me?
Pre project interviews with each pilot grantee Data collected in April 2023 (*n* = 3)	• Tell me what wellness means to you and your community. • Tell me about your expectations of NAME OF PROGRAM. ○ Probes: content, activities, length, challenges • Tell me about your anticipated challenges of NAME OF PROGRAM ○ Probes: time, recruitment, resources • If you had to explain NAME OF PROGRAM to a community member, what would you say? ○ Probes: content, activities • If you had to explain NAME OF PROGRAM to a funder, what would you say? ○ Probes: resources needed, content, activities, priorities • Please tell me about your experience with recruitment and retention of participants in NAME OF PROGRAM in your community. ○ Probes: challenges, strategies, recommendations • Tell me what you think about SNAP-Ed funding to finance your NAME OF PROGRAM. ○ Probes: challenges, strategies, recommendations • If you had to explain nutrition education in your community to someone outside of your community, what would you say? • Is there anything else about NAME OF PROGRAM that you would like to share with me?
Post project interviews with each pilot grantee Data collected in November 2023 (*n* = 2)	• Tell me about your experience with the NAME OF PROGRAM. ○ Probes: content, activities, length, challenges • If we were to change anything about the NAME OF PROGRAM, what would it be? ○ Probes: content, activities, logistics • If there was anything that you really liked about NAME OF PROGRAM, would you tell me about that? ○ Probes: content, activities • Help me understand your thoughts on continuing this NAME OF PROGRAM in your community. ○ Probes: resources needed, time, funding, concerns • Please tell me about your experience with recruitment and retention of participants in NAME OF PROGRAM in your community. ○ Probes: challenges, strategies, recommendations • Tell me what it was like using SNAP-Ed funding to finance your NAME OF PROGRAM. ○ Probes: challenges, strategies, recommendations • If you had to explain SNAP-Ed funding to another community organization, what would you say it was like? ○ Probes: challenges, strategies, recommendations • Is there anything else about NAME OF PROGRAM that you would like to share with me?

### Request for Proposals development methods

To solicit Indigenous-serving organizations in Colorado interested in applying for SNAP-Ed pilot funding, faculty, and staff at CAIANH drafted an RFP that was guided by the listening sessions and by the SNAP-Ed guidance. Three Indigenous collaborators reviewed the RFP and provided guidance regarding language that was overly “academic” and feedback as to requirements and application process instructions. Based on these reviews, a 2-page RFP was sent to the 8 organizations who participated in the listening sessions. The listening sessions-informed RFP included: background on USDA SNAP-Ed, eligibility (e.g., participated in phase 1 listening session), and detailed application instructions (e.g., title page, 2-page narrative, 1-page timeline, 2-page budget, and justification). It was not required that eligible organizations had previous experience with SNAP-Ed funding and applicant organizations were invited to propose activities they considered “nutrition education” for their communities. Of note, which activities were ultimately funded depended on SNAP-Ed guidance and negotiation with State SNAP-Ed leadership. The RFP also included details on the review process, submission guidance, and key deadlines. The RFP also delineated that grantees would be requested to participate in pre-/postinterviews with the CAIANH-based research team as part of this case study evaluation. Three of the 8 eligible organizations applied for pilot funding, and all 3 were awarded the full budget as they requested. Details of the 3 organizations can be found in Table 2.

**TABLE 2:** Details on the 3 Indigenous-serving organizations that participated in Colorado SNAP-Ed pilot funding project.

Organization Name(s) and Location	Overview of organization	Overview of SNAP-Ed project
The Acequia Institute and The San Luis Peoples Market Located in the San Luis Valley, in Southeastern CO	The Acequia Institute (TAI) was founded in 2005 and incorporated in 2007 as a 501(3) nonprofit organization headquartered in Seattle, WA. In 2006, TAI acquired a historic 181-acre acequia farm on lands deeded in 1852 under the Sangre de Cristo Land Grant of 1844 and initiated a new headquarters presence in CO, where the land was managed as an agroecology research farm and ecological restoration project. Since 2007, TAI nonprofit programs have included small direct to producer grants, tuition scholarships for local youth from acequia farm families, fellowships to Native American and other BIPOC graduate students involved in environmental justice or food sovereignty research, food justice conference sponsorships, funding for seed exchange networks, and many other charitable donations like our annual “firewood for elders” campaign. TAI has established an endowment to support a mutual aid revolving loan fund offering no-interest loans for local BIPOC women producing artisan foods for sale at the San Luis Peoples Market and for use in SNAP-Ed cooking and nutrition classes.	This SNAP-Ed project is entitled *Decolonizing Chicanx Diets: Nixtamal, Traditional Food Crops, and Heritage Cuisine as Interventions to Address the Endemic Diabetes and Obesity Crisis in Costilla County, CO.* The proposed project introduced a decolonial culinary and nutritional program focused on the revival of the production, preparation, and consumption of traditional crops and a return to heritage cuisines among the priority population of local (e.g., rural-dwelling) Indigenous youth in our farm internship program and SNAP and eWIC recipients. The cooking class component of the project took place at the San Luis Peoples Market commercial kitchen or other appropriate venues.
Spirit of the Sun, INC (SOTS) Located in Denver, CO	SOTS’s mission is to work in partnership with Native American communities in urban areas and on reservations to boost resilience of Native people, especially youth and young adults. In operation since 2002, one of the main priorities of this organization is to address food insecurity in their priority urban-based community.	Nutrition education and food security programs supported by SNAP-Ed included Elders Foodshare, Indigenous Agriculture, Outdoor Program, Mycelium Healing Project, and Indigenous Science and Foodways Youth Leadership Program. This program serves Native/Indigenous youth in Denver and surrounding areas, and youth leaders learn about Indigenous foodways, heirloom seed preservation, herbal and medicinal landscapes, and growing vegetables and fungal produce to distribute to elders and families through the Elder’s Foodshare program. SNAP-Ed also supported SOTS’s mission to provide culturally relevant food to Native and Indigenous communities.
Haseya Advocate Program Located in CO Springs, CO	Haseya Advocate Program is currently the only response for urban Native survivors of gender-based violence in CO. All of their services are free and culturally centered, and they help survivors heal from trauma through their Indigenous teachings. One of the least intimidating ways to address cultural teaching is to focus on food. They do this by fostering a connection to the land and water while talking about our food being medicine and how the intentional destruction of our traditional food systems was a control tactic. They regularly discuss how we can learn language and ceremony through our traditions related to food harvesting and preparation. Survivors gain empowerment through these teachings, and they are also helping to meet their families’ basic need for healthy food in the process. Priority population includes Indigenous domestic violence survivors from both urban and rural locations.	SNAP-Ed supported implementing varying levels of food access and food education. Haseya includes land and water-based teachings such as kayaking, hiking, gardening, urban foraging, fishing, and butchering. Haseya manages *Piath Ket Naa Nath*, an Indigenous healing garden, where survivors can come to garden, learn Native plant identification and usage, and participate in ceremonies or seasonal celebrations. This grant allowed Haseya to purchase several kitchen items to be able to implement a food preservation component so they can teach canning, dehydration and storage of excess produce. They learned that many of the families served did not grow up eating in a traditional or even nutritious way so this started healthier eating habits for the community. Haseya hosted cooking instruction in the demonstration kitchen and hosted a local Odawa/Diné chef to teach some of those classes.

BIPOC, Black, Indigenous, and people of color; eWIC, electronic Women Infants and Children.

### Data collection

Listening sessions and pre-/postinterviews with pilot grantees were conducted using semistructured moderator guides (Table 1). The listening sessions were moderated by a non-Indigenous researcher with expertise in qualitative methods and with an Indigenous notetaker. The listening sessions were not recorded per the preference of the participants. The pre-/postinterviews with pilot grantees were recorded and were conducted by a non-Indigenous collaborator with no notetaker present. Listening sessions were conducted from October to November 2022 (*n* = 8), preinterviews in April 2023 (*n* = 3), and postinterviews in November 2023 (*n* = 2). Although all pilot grantees participated in the preinterviews, one of the pilot grantee organizations was unable to participate in the postinterview due to scheduling conflicts.

### Analysis

Pre-/postinterviews were recorded, and all audio recordings were professionally transcribed verbatim. The lead qualitative researcher checked each transcript for accuracy against the audio recordings. Next, researchers used Atlas.ti as a digital qualitative management tool to facilitate organization, coding, and memo-ing the data [13]. The interview transcriptions were coded using qualitative content analysis methods [14,15], which helped compare transcripts to understand salient cross-case themes. To honor the storytelling approach [11], data were coded by the research team in various quotation increments depending on context, as line-by-line coding did not support the epistemologic view of the researcher [16]. The first pass coding involved inductive free coding, as supported by a constructivist approach, which was narrowed by collapsing and integrating codes for redundancy during the second pass, which involved describing and defining each code [16]. An example includes codes “foraging,” “seed keeping,” and “soil health,” which supported development of the category “traditional foodways,” which then supported the first theme. All authors of this paper participated actively in study design, data collection, data analysis, and/or writing this manuscript, 4 of whom identify as Indigenous or American Indian. As a means to redistribute power, all pilot grantee leadership were invited to serve as coauthors on this manuscript. The funding organization’s leadership did not participate in data collection, analysis, or interpretation of results.

## Results

### Listening sessions

During October and November 2022, 8 listening sessions were conducted with 8 different Indigenous-serving organizations in CO. The listening sessions were held via Zoom, and for each of the 8 listening sessions, between 1 and 4 individuals from each organization attended (15 individuals in total). Listening sessions ranged from 34 to 78 min in length. Findings from the listening sessions (*n* = 8) indicated that there are many ongoing food and nutrition education programs serving Indigenous people in CO. Examples include education and community-based gardening, cooking, traditional food acquisition, and food preservation. This programming is largely to promote wellness and healing through traditional cultural practices (including foodways) and not necessarily called “nutrition education” or overtly framed as nutrition education. Rather, much of this programming focuses on connecting community to traditional cultural practices, mental health support, skills building/job training, parenting support, traditional physical activity, and largely to support Indigenous community healing and holistic well-being. Existing programming is informed by Indigenous ways of knowing (compared with hypothesis-tested, evidence-based) and does not typically follow a “curriculum” as Western education systems might require. These examples offer key opportunities on how to Indigenize nutrition education (e.g., transform teaching practices to center Indigenous knowledge) and promote both strength- and assets-based programming (e.g., focus on healthful traditional cultural ways). Finally, a key finding is that most existing nutrition education-based programming for Indigenous peoples in CO is led and facilitated by Indigenous educators.

### Pilot project interview findings

In April 2023, Indigenous leadership from each of the pilot organizations engaged in a 1:1 storytelling-based preinterview [11,12] with a non-Indigenous interviewer via Zoom [17]. These interviews ranged from 55 to 69 min in length [18]. Two of the 3 pilot grantee organizations participated in a 1:1 postinterview with the same researcher via Zoom. These interviews ranged from 62 to 77 min in length and took place in November 2023. Two salient themes emerged from the pre-/postinterviews with the pilot organizations. The first theme includes defining and describing indigenized, decolonized, and culturally grounded nutrition education. The second theme includes description of experiences working with SNAP-Ed funding during this pilot project, including challenges and future opportunities. Of note, the “participants,” as referenced in the next sections of this manuscript, are the leadership and implementers of SNAP-Ed funded food and nutrition programs in Indigenous communities in CO.

#### Theme #1 – Describing and defining indigenized, decolonized nutrition education

All participants shared that, first and foremost, their nutrition and food-based programming is rooted in Indigenous values, land-based, fosters holistic health and healing, and focuses on interconnectedness and relationality. One participant explained:

“We’re here to provide Indigenous foods to Indigenous people. And like I said before, make it accessible, normalize it, and not just provide access to the food, but also teach how to use the food, how to cook it, how to prepare it, how to preserve it, how to store it. (…) So, it’s food for holistic healing. So, food is part of the holistic healing component of our program.” (Organization A)

Some participants discussed the importance of intergenerational learning—by connecting youth and elders and focusing on traditional food acquisition such as foraging, seed keeping, canning, dehydration, and traditional food preparation and recipes. They talked about what nutrition education is not—as described by another participant:

“Nutrition education. I think we need to focus more or less on food labels and like not necessarily from a registered dietician kind of standpoint, (…) Native people have been scientists for thousands of years. More about our relationship with our food, whether that is some of the mental health stuff that comes along with relationships with food, but also, are you living seasonally? Do you know where your food is coming from? Are you part of that process? Are you putting down tobacco and paying respects to that food? Do you know how to cook the food that your great-grandma ate? (…). And so our food education is less about a food pyramid and more about our traditional pathways that go on how we obtain and how we use our food, and having less waste and being more intentional about it, and really looking at the way our people have been thriving for thousands and thousands of years with that relationship with our land and our food and getting back to some of that in ways that it’s reasonable to reincorporate back into our lifestyles. (…) Food education is language education. It’s ceremonial education. It’s living within our seasons education. It’s not just the food. The food is the end result of all those other things. And so how do we have intentional relationships with our food as Native people that we can go back to nourishing our bodies in a way that (…) We have to reestablish our relationships with those plant relatives and the animal relatives and realize we need them, they don’t need us, and approaching that in a humble way. (Organization B)

Some participants also discussed the importance of Indigenous-led education and programming. They illustrated how some Western-based models of nutrition education will not authentically reach their communities, as discussed here:

“So, the nutrition is there, and a lot of that is going to be in our Indigenous cooking classes, but a lot of it will not be in written form. So, I don’t know for SNAP [Ed] what the requirement is, but we did voice our concerns that if people really want to work with Native and Indigenous peoples, things need to be changed on those policies on the front end rather than... Even the pyramid. We don’t like using the pyramid because we’re not hierarchal. We’re trying to move to a lateral circle and a lateral organization. But a lot of it is written by white males. And so how do we change that even to what we call ‘rematriate,’ to have female leadership, to be working under females or working with more females and BIPOC folks.” (Organization C)

Another participant specifically shared his perspective on the relationship between Indigenizing food systems and decolonization:

“The violence of settler colonialism, including territorial dispossessions and ecological despoliations, resulted in the destruction of the material basis of food self-sufficiency of the Tribal nations and other Indigenous peoples. The practice of *re-indigenizing* food and foodways is therefore completely tied up with resistance to the imposition of colonized diets or decolonization. (…) Thus, *re-indigenizing* our food systems, both agroecological and culinary dimensions, involves resistance against colonization of our diets and foodways. The *two* are inextricably interwoven.“ (Organization A)

Finally, all participants discussed the innate sustainability of traditional foodways and how colonization impacted Indigenous people’s ability to actualize and practice their cultural traditions. They all underscored the importance of land in terms of land access and honoring the Indigenous value of mutual reciprocity and relationality with the land. Much of the food and nutrition education is land- and water-based and inherently includes hands-on, interactive learning as a key traditional way of knowing. All participants emphasized the effects of settler colonial violence on both the land and Indigenous peoples, explaining that both (land and people) have experienced intergenerational historical trauma. This is a key reason why farming and gardening and other land-based education activities are central to indigenized nutrition education. An example of how “nutrition education” is operationalized is a regional food system where the central tenant is that the health of the soil (e.g., land) is directly related to human health—often called the “dirt-to-gut” model [[19], [20], [21]]. Some of the organizations’ youth interns learn how to grow crops and manage soil health, and then they learn to prepare and cook the crops they grew in the cooking and nutrition classes, fomenting a holistic dirt-to-gut-and spirit learning experience.

#### Theme #2 – Experience with SNAP-Ed pilot funding mechanism: challenges and future opportunities

Participants shared key challenges in engaging with SNAP-Ed funding, which included sentiments of bidirectional mistrust (e.g., grantee of funder and funder of grantee) and of community members in participating in “government-funded” programs. One key solution to mitigate this mistrust is to work with Indigenous collaborators from all parties involved (e.g., funders and grantees). They also discussed the challenges inherent to working at small organizations (e.g., small number of staff and small budget) and that this might lead to a smaller “reach, but they would prefer to focus on the quality of outreach rather than quantity of individuals reached. Additionally, evaluation metrics, as required by “most funders,” are not usually aligned with Indigenous values and ways of knowing. One participant explained:

“I would say that we are taking back our traditional food ways in a way that is community-driven so that the community is deciding what it is they want us to do. If you’re going to be funding us, then trust that we have the capacity to do this and that we will do it in a way that the Native people want us to do. So, it might not look the same as the data and the metrics that they want, but we can measure our success in different ways through narratives, *through* photo stories, impacts on specific families. So, we might not be serving thousands of families. Actually, we will not be serving thousands of families, but the impact that we’re going to have on the families that we do serve is probably going to be much more significant and hopefully changing behaviors *on a* long-term when it comes to food and cooking than other food pantries do where they just provide food.” (Organization B)

They also said that, with limited staff and capacity, it can be very challenging to work with a reimbursement funding system, and they suggested that this is “an example of how the government doesn’t trust us.” Finally, they suggested more time (e.g., longer funding cycle than 7 mo provided in this pilot project) and flexibility with “unallowable” costs should be considered for their unique programming. One participant explained what funders need to take into consideration when engaging Indigenous communities:

“And so we understand that we have to do this dance, but I’m not going to do the dance in order to get $100,000, and then we have to do it the way that SNAP-Ed wants us to do it. And so I would love to see some minor changes and have them *to* be open and receptive to some of the things that we need that would be in the best interest of our communities. So I think what I’m trying to say is I don’t mind being on calls and asking for money, but we just can’t change what we’re doing. (…) It’s almost like the reciprocal relationship building too, because I don’t want this to be just a one-year thing. I want this to be long-term. I want people from your organizations *and* circles and leadership spaces to understand it. But from my feelings coming from a leader *is*, we’re hoping that those folks will think about it as well of what they’re doing, wanting to intentionally do for our Native community and not put so many more rules and policies on how we need to use this money because we need to do it the way we need to do it for our own communities.”(Organization A)

Participants also discussed successes and opportunities based on their experience as pilot grantees. First, they appreciated the relationships built with the Colorado SNAP-Ed implementing agency leadership at the University of Colorado Anschutz Medical Campus and the flexibility allowed in this pilot funding cycle. They suggested the flexibility in program implementation, budget, and evaluation is well aligned with authentic efforts to engage Indigenous communities. One participant shared:

“SNAP-Ed funding was utilized in different areas of support for food security, food sovereignty, community-building, education, training, really looking at other areas that we haven’t had before with other funding. It was flexible. I mean, to me, it seemed really flexible. Working with you guys is really just awesome because we don’t get this much funding or funder attention, working with the program officers and the coordinators. We don’t get that with other (…) like SNAP-Ed funding are very attentive, attentive, and listening. We don’t get a lot of funders like that. Most calls are like, “Okay. We’re going to do this, blah, blah, blah,” and then they’re off in 5 or 10 minutes or 15 minutes tops. They’re just breezing through everything. (…) I’m really grateful that SNAP-Ed, everyone who has participated has been very, very attentive, and intentional. I think that really counts for something, not that we’re just there and then they’re just going to push us out of the way. It’s almost like we’re in line and then you’re getting served, and then that’s it. But I feel that way. (…) in communication with you as much as me, but just that personal development of relationship building, that’s key for me.” (Organization A)

Participants shared the importance of relationship building and especially appreciated opportunities to meet University-based staff in person, such as at the annual Conference on Native American Nutrition in Minnesota and inperson site visits. Finally, participants shared hope this pilot funding cycle will be continued, and that collaboration and bidirectional learning can ensue in years to come.

## Discussion

These findings elucidate the experiences, challenges, and guidance for advancing SNAP-Ed within Indigenous communities. To effectively address the priority area for USDA Food and Nutrition Service SNAP-Ed funding to reach Indigenous communities in each state, understanding the perspectives of Tribal and Indigenous-led communities new to SNAP-Ed funding is prudent. Several Indigenous-serving organizations and Tribes have effectively used SNAP-Ed funding for many years, including social media nutrition education [22], family- and school-based cooking and nutrition education [[23], [24], [25]], and youth-focused type 2 diabetes prevention programming through traditional storytelling [26,27]. Recognizing these successful SNAP-Ed-funded programs in Tribal and Indigenous communities sets precedent for actualizing USDA’s new SNAP-Ed guidance and provides successful model examples of SNAP-Ed implementing agencies working with Tribal communities. Additionally, publications from First Nations communities in Canada provide foundation for research on decolonized diets and food systems [28], decolonized food literacy education [29], and indigenized food sovereignty [30].

This study examined the experiences of Indigenous-led organizations new to SNAP-Ed funding. Diversity, equity, inclusion, and justice efforts across the United States use terms such as “decolonize” and “Indigenize,” but in many cases, there is paucity in how these concepts are actualized [31]. We posit that re-Indigenizing food systems is a form of resistance against colonization of foods and foodways (e.g., decolonizing). Nutrition and food education provide tangible examples of how these concepts can be operationalized. Expanding definitions of nutrition education is a key approach to operationalizing concepts such as Indigenizing, self-determination, sovereignty, and decolonization. In terms of food and nutrition education, these concepts can be used as examples of how other health promotion and disease prevention programs can better align their emphasis, content, and delivery to Indigenous people. For example, grantees in this CO-based pilot project largely focused on healing communities by connecting youth and community members to their traditional foods—whether through improving access, teaching about foraging, seed keeping, and other ways of acquiring traditional foods, or cooking and preparing traditional foods. Although “nutrition education” was not the overt goal of any given pilot grantee, the salient nature of food and nutrition, as it applies to all community members (e.g., elders, youth, and those with chronic disease), is a tangible way to reintroduce traditional cultural practices into the lives of those for whom these practices have been forcibly devastated through the historical impacts of colonization.

As expected, the pilot grantees experienced some challenges in working with SNAP-Ed funding. One goal of this pilot funding cycle was to identify these challenges and elevate concerns to state-based program administrators and federal funders. Many of the challenges are related to the small size of organizations, who may not have the financial or administrative infrastructure to provide monthly invoices or the financial backing to operate within the SNAP-Ed reimbursement model. These pilot organizations are small in nature (e.g., <5 full-time staff members), and given the relatively small numbers of Tribal or Indigenous people living in and across CO, concerns about “getting enough” participants at any given cooking class or nutrition/food program were persistent. Arguably, smaller organizations are, therefore, at a further disadvantage from accessing and engaging with federal funding such as SNAP-Ed, yet these small organizations often serve communities (e.g., rural) that are consistently left out of such funding opportunities.

Grantees shared concerns about the source of funding (e.g., federal government) and the source of food (commodities); not surprisingly, then, they much preferred to work with Indigenous collaborators, funders, food producers, and food suppliers. Because of a long history of broken treaties, racist policies, and systemic oppression of Indigenous peoples, rebuilding and establishing trust has long been a dilemma for federal agencies and other non-Tribal/Indigenous collaborators in working with these communities [26,27,32,33]. One strategy the University of Colorado SNAP-Ed implementation agency employed to build trust and foster open communication was to hire an Indigenous consultant, who is an expert in food and nutrition among Indigenous communities and also familiar with SNAP-Ed guidance, to serve as a liaison between pilot grantees and University-based researchers and staff. Another strategy to build trust, as supported by Colorado SNAP-Ed leadership, was to operate as flexibly as possible regarding allowable costs, programming requirements, and evaluation deliverables. As detailed in the 2013 Roadmap for Collaborative and Effective Evaluation in Tribal Communities, there are many strategies to evaluate programs that reach Tribal communities—all rooted in building trusting relationships, elevating Indigenous values and ways of knowing, and focused on building a new narrative about what effective evaluation and outcomes look like [34].

Key limitations of this project are related to the unique nature of this pilot study and the fact that findings may not be generalizable to other SNAP-Ed-funded organizations. For instance, all of the pilot grantees identify as Indigenous, as do all of their educators. Other SNAP-Ed-funded programs that do not have Indigenous leadership and staff may face different challenges to collaborating with and serving Indigenous communities. One way to mitigate the lack of Indigenous nutrition educators in any given community would be to expand who is “credentialed” to be a nutrition educator and to center the importance of cultural competency and place-based Indigenous knowledge just as important as formal “nutrition education” training. Another limitation is that no Tribes, Tribal organizations, or communities who use the Food Distribution Program on Indian Reservations were included in this pilot project, so the generalizability may be insubstantial to these groups. A key strength of this project is the continued trust-building, bidirectional learning, and cocreative process between Indigenous and non-Indigenous researchers.

Next steps to the continued cocreation of SNAP-Ed opportunities in Indigenous spaces include educating funders and policymakers about Indigenous ways of knowing, traditional food and nutrition practices, and importance of relationality, land, healing, and other Indigenous values. Determining the “success” of a program through program evaluation needs to be informed by the community the program serves, and for Indigenous communities, the unit of analysis might not be a single individual person’s “knowledge” (e.g., as assessed in pre-/postsurveys) but rather focus on relationality with land and land- (or water-) based resources. Further, intended outcomes might not center exclusively on an individual person’s health but focus more broadly on community health, relationships (e.g., with land and nonhuman relatives), or other nonhealth-specific outcomes as prioritized by community. Finally, elevating the voices of Indigenous peoples to equitable roles in conversations about policies related to SNAP-Ed (or other funding mechanisms) is a key strategy to Indigenize the funding mechanism as a whole and benefit all concerned.

## Author contributions

The authors’ responsibilities were as follows – SM, SAS, JP: designed the research; SAS, KB, DP, SF: conducted the research, SAS analyzed the data; SAS, JP, SM: wrote the paper and had primary responsibility for the research and all authors: read and approved the final manuscript.

## Funding

This study was supported by Colorado SNAP-Ed (IHGA # 177430) PI: Puma; NIDDK (5K01DC128023) PI: Stotz; NIDDK (P30DK092923) MPI: Manson.

## Data Sharing

Data described in this manuscript will be made available upon request, pending consensus approval from all coauthors.

## Conflict of interest

All authors report no conflicts of interest.
